# Quantification of Water, Protein and Soluble Sugar in Mulberry Leaves Using a Handheld Near-Infrared Spectrometer and Multivariate Analysis

**DOI:** 10.3390/molecules24244439

**Published:** 2019-12-04

**Authors:** Yue Ma, Guo-Zheng Zhang, Sedjoah Aye-Ayire Rita-Cindy

**Affiliations:** 1 School of Biotechnology, Jiangsu University of Science and Technology, Zhenjiang 212018, China; mayue15751773676@163.com (Y.M.); ritacindy19@gmail.com (S.A.-A.R.-C.); 2 Sericultural Research Institute, Chinese Academy of Agricultural Sciences, Zhenjiang 212018, China

**Keywords:** mulberry leaves, water content, crude protein, soluble sugar, near-infrared spectroscopy, hand-held spectrometers, wavelength selection

## Abstract

Mulberry (*Morus alba* L.) leaves are not only used as the main feed for silkworms (*Bombyx mori*) but also as an added feed for livestock and poultry. In order to rapidly select high-quality mulberry leaves, a hand-held near-infrared (NIR) spectrometer combined with partial least squares (PLS) regression and wavelength optimization methods were used to establish a predictive model for the quantitative determination of water content in fresh mulberry leaves, as well as crude protein and soluble sugar in dried mulberry leaves. For the water content in fresh mulberry leaves, the R-square of the calibration set ($R_{C}^{2}$), R-square of the cross-validation set ($R_{CV}^{2}$) and R-square of the prediction set ($R_{P}^{2}$) are 0.93, 0.90 and 0.91, respectively, the corresponding root mean square error of calibration set (RMSEC), root mean square error of cross-validation set (RMSECV) and root mean square error of prediction set (RMSEP) are 0.96%, 1.13%, and 1.18%, respectively. The $R_{C}^{2}$, $R_{CV}^{2}$ and $R_{P}^{2}$ of the crude protein prediction model are 0.91, 0.83 and 0.92, respectively, and the corresponding RMSEC, RMSECV and RMSEP are 0.71%, 0.97% and 0.61%, respectively. The soluble sugar prediction model has $R_{C}^{2}$, $R_{CV}^{2}$, and $R_{P}^{2}$ of 0.64, 0.51, and 0.71, respectively, and the corresponding RMSEC, RMSECV, and RMSEP are 2.33%, 2.73%, and 2.36%, respectively. Therefore, the use of handheld NIR spectrometers combined with wavelength optimization can fastly detect the water content in fresh mulberry leaves and crude protein in dried mulberry leaves. However, it is a slightly lower predictive performance for soluble sugar in mulberry leaves.

## 1. Introduction

Mulberry is a perennial root plant, and the leaves contain a variety of nutrients, such as proteins, soluble sugars, and fat, which are essential nutrients for the growth and development of silkworm [1]. The amino acids (made up of proteins) in mulberry leaves are abundant and suitable in proportion, and the essential and semi-essential amino acids account for more than half of the total amino acids, with the contents of methionine and lysine higher than that of conventional feed. Besides, mulberry leaves are palatable, highly digestible, and barrier-free feeding. They contain a variety of biologically active substances, which have effects on the improvement of immunity, anti-inflammatory, and anti-oxidant [2,3].

In the traditional sericulture industry, mulberry leaves are mainly used as feed for silkworms [4]. In recent years, mulberry leaves have been extended from silkworm feed to livestock feed. Islam [5] confirmed that the addition of mulberry leaf meal to broiler feed could lower their cholesterol and reduce production costs. Zhu [6] added 15% mulberry leaf powder to the finished pigs’ diet and found that it can change the muscle fiber properties, resulting in enhanced antioxidant capacity and increased intramuscular fat to improve meat quality. At present, the demand of mulberry leaves is increasing in the livestock industry [7]. However, differences in a mulberry growth environment and field management can result in quality differences of mulberry leaves [8]. In order to obtain high-quality mulberry leaves, a fast, simple, and effective method for the determination of mulberry leaves is in urgent need, instead of the conventional wet biochemical methods that have disadvantages of a long time, high cost and inconvenient.

NIR spectroscopy is widely used in food for advantages rapid detection, low analysis cost, excellent reproducibility [9,10,11]. Toledo-Martín et al. [12] used NIR spectroscopy and modified partial least squares (MPLS) to establish a regression model for the fast determination of total phenolic content (TPC) and total carotenoid content (TCC) in blackberry. The ratio of the standard deviation to standard error of prediction (performance) (RPD) and ratio of the range to standard error of prediction (RER) of the TPC model were 1.52 and 5.92, respectively, and the RPD and RER of the TCC model were 1.82 and 8.63, respectively. The results show that NIR spectroscopy can be used for the detection of substances in blackberries. In recent years, NIR spectroscopy has also been used in the feed industry [13,14,15]. Swart et al. [16] successfully used NIR spectroscopy to detect dry matter (DM), ash, crude protein (CP), ether extract (EE), crude fiber (CF), acid detergent fibre (ADF), neutral detergent fibre (NDF), gross energy (GE), calcium (Ca), phosphorus (P), etc., to achieve a rapid, non-destructive quantitative analysis of nutrients in the ostrich mixed rations. Tahir et al. [17] used NIR reflectance spectroscopy to achieve accurate estimates of total and phytate phosphorus in poultry feed.

The recent progress in miniaturization that has taken advantage of new micro-technologies such as micro-electro-mechanical systems (MEMS), micro-opto-electro-mechanical systems (MOEMS) and micro-mirror arrays or linear variable filters (LVFs) has led to a drastic reduction of spectrometer size while allowing excellent performance due to the high-precision implementation of essential elements in the final device, which is dramatically facilitates the on-site and real-time detection [18]. Neve et al. [19] applied hand-held NIR instruments to record the NIR spectra of a variety of different pasta sauce blends and established six models for different nutritional parameters such as energy, protein, fat, carbohydrate, sugar and fiber. The predictive model, the experimental results show the feasibility of handheld NIR spectroscopy to predict dietary nutrition parameters.

Thus, based on the handheld NIR spectrometer, this study established rapid analysis methods for the determination of water content in fresh mulberry leaves, and crude protein and soluble sugar in dry mulberry leaves. Three wavelength optimization methods, including uninformative variable elimination (UVE) [14], competitive adaptive reweighted sampling (CARS) [20], and random frog (RF) [21] were used to select high informative wavelength variables to improve the determination accuracy.

## 2. Results and Discussion

### 2.1. Spectral Characteristics

**Figure 1:**
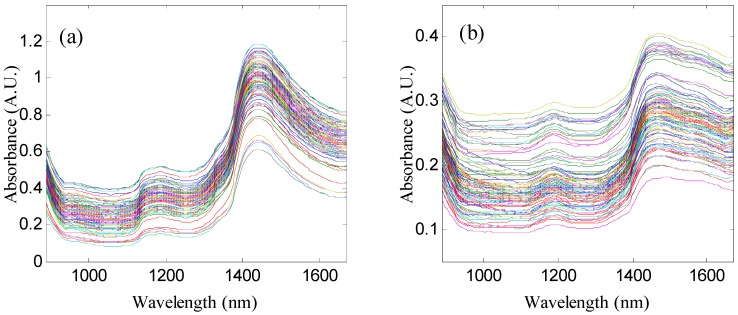
Raw spectra of: (**a**) fresh mulberry leaves, (**b**) mulberry leaf powders.

The raw NIR spectra of fresh mulberry leaves and dry mulberry leaves are shown in Figure 1, which shows that the primary trend of the spectral curves is similar. The spectra have an overtone absorption peak of the weak C-H bond at 1190 nm and a distinct -OH absorption peak at 1440 nm. The peak strength of -OH bond absorption peaks in the spectra of fresh mulberry leaves is higher than that of dry mulberry leaves, which is mainly due to the higher water content in fresh mulberry leaves.

### 2.2. Reference Values

The statistics of water content, crude protein, and soluble sugar in mulberry leaves are shown in Table 1. The range of water, crude protein, and soluble sugar in mulberry leaves were 60.44~78.46%, 11.10~23.50 and 8.47~31.01%, the average and standard deviation were 68.24 ± 3.75%, 17.41 ± 2.27%, 19.97 ± 3.92. The range, average, and standard deviation of the calibration set and the unknown sample set are close, indicating that these values are highly representative, so the model constructed will be better applied in practice.

### 2.3. Spectral Pretreatment

**Figure 2:**
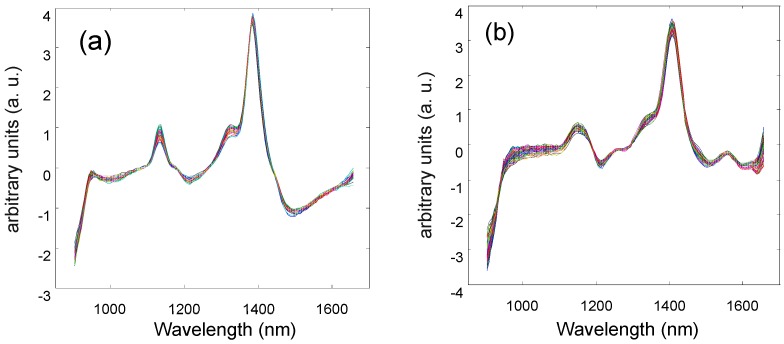
The spectra pretreated with 1st Der+ SNV+ autoscaling for: (**a**) fresh mulberry leaves, (**b**) dry mulberry leaves.

Different methods were used to pretreat spectral data. The results are shown in Table 2, in which the pretreatment has significantly affected the prediction accuracy of the models. The combination of the first-order derivative (1st Der) + standard normal variate (SNV) + autoscaling pretreated spectra show the best results of modeling (Figure 2).

As shown in Table 2, for the water content, when the optimal number of factors is seven, the $R_{C}^{2}$ and $R_{CV}^{2}$ are 0.92 and 0.90, respectively, and the corresponding RMSEC and RMSECV are 1.00% and 1.17%, respectively. When eight factors are applied for the protein content, the $R_{C}^{2}$ and $R_{CV}^{2}$ are 0.90 and 0.83, respectively, and the corresponding RMSEC and RMSECV are 0.74% and 0.97%, respectively. For soluble sugars, when the optimal number of factors is seven, the $R_{C}^{2}$ and $R_{CV}^{2}$ are 0.60 and 0.45, respectively, and the RMSEC and RMSECV are 2.45% and 2.90%, respectively. The pretreatment of the raw spectra improves the prediction accuracy of the model because SNV can correct the scattering caused by sample roughness and particle unevenness, the first derivative can deduct the baseline drift and background noise interference to improve resolution, and autoscaling enhances the difference between spectral data [15].

### 2.4. Wavelength Optimization

**Figure 3:**
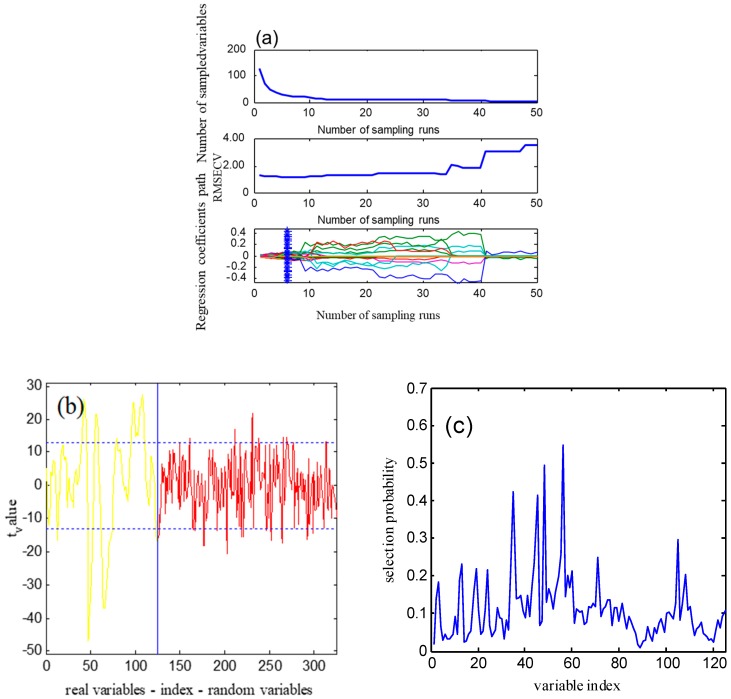
Wavelength variable selection with the: (**a**) CARS, (**b**) UVE, (**c**) RF.

Figure 3 shows diagrams of the wavelength variable screening for the water content of mulberry leaves. For the CARS (Figure 3a), the first graph is the trend graph of the number of selected wavelength variables with the number of sampling runs. As the number of sampling runs increases, the number of selected wavelength variables decreases from fast to slow. 

The second graph is a graph of RMSECV changes. Before the sample was iterated seven times, RMSECV gradually decreased, indicating that the wavelength variables not related to the moisture content of mulberry leaves were eliminated. After seven times, RMSECV gradually increased, indicating that essential wavelength variables related to the moisture content of mulberry leaves were eliminated. The third graph is the changing trend of the regression coefficient of each wavelength variable during the screening process. The position of “*” in the figure corresponds to the minimum value of RMSECV. The colored line indicates the trend of the regression coefficient of each wavelength variable, which increases as the number of samples increases.

For the wavelength selection of UVE, as shown in Figure 3b, data on the left side of the abscissa is the actual spectral wavelength variable, and the right part is the system’s noise variable generated by the random noise simulation. The numerical values in the ordinate direction indicate the stability of each wavelength variable, and the two horizontal dashed lines represent the stability threshold of the selected actual spectral wavelength variable. The wavelength variable corresponding to the stability value within the threshold range didn’t participate in PLS modeling. The stability variables outside the threshold range were useful for the water content of mulberry leaves and were selected for the PLS modeling.

For the RF, as shown in the wavelength variable screening graph (Figure 3c), the ordinate is the probability of the wavelength variable selected. According to the importance of the wavelength variable, 50 wavelength variables with larger possibilities were selected to participate in the PLS modeling.

**Figure 4:**
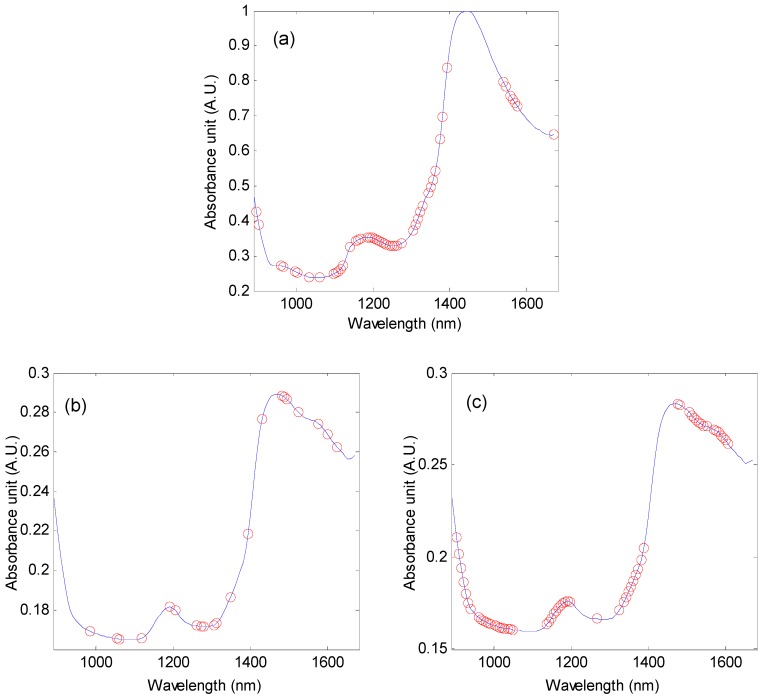
The selected wavelength variables for: (**a**) water content in fresh mulberry leaves, (**b**) crude protein in mulberry leaves, and (**c**) soluble sugar in mulberry leaves.

The selected wavelength variables are shown in Figure 4. For the determination of water content in fresh mulberry leaves, it is interesting that the selected wavelength variables are not shown in the absorption peak of water, and the variables on the shoulder were selected. For the protein, a few variables are selected. For the soluble sugar, the selected variables are similar to that for water, which may be that a lot OH in soluble sugar.

The number of factors has a significant impact on the prediction ability of models. When the number of factors is less, it does not reflect the characteristics of the substance, which leads to a low prediction accuracy of the model. Many factors lead to over-fitting, which gives a high prediction accuracy; however, when applied to unknown sample detection, the prediction effect is weak. In this work, the cross-validation of leave-one-out was applied to obtain an optimal number of factors. The results are shown in Table 3.

For the water content in fresh leaves, the RF method has the best performance in the selection of wavelength variables, and the final wavelength variables were reduced from 125 to 50 (Figure 4a). The RMSEC and RMSECV are 0.96% and 1.13%, respectively, and decrease by 4.00% and 3.42%, respectively, compared to the model with the whole wavelength variables. The corresponding $R_{C}^{2}$ and $R_{CV}^{2}$ are 0.93 and 0.90, respectively, Cross-validation relative analysis error (RPDCV) was 3.25. The low RMSEC and RMSECV values, and the high $R_{C}^{2}$ and $R_{CV}^{2}$ values indicate the high prediction accuracy of the model. Furthermore, their values are similar, which demonstrates that the model is robust. Therefore, the model can be accurately and reliably used to predict the water content of unknown mulberry leaves.

For crude protein, the CARS, UVE, and RF methods effectively improved the prediction accuracy of the model, among which the CARS method was the best (Figure 4b). When the number of optimal factors is 9, the $R_{C}^{2}$ and $R_{CV}^{2}$ are 0.91 and 0.83, respectively, and the corresponding RMSEC and RMSECV are 0.71% and 0.97%, respectively, and the RPDCV is 2.43, it indicates that the model can predict crude protein of mulberry leaves. However, the difference between RMSEC and RMSECV values indicates that the model is less robust.

For soluble sugar, 60 spectral wavelength variables were selected to establish the PLS model (Figure 4c). When the optimal factor is 8, the RMSEC and RMSECV are 2.33% and 2.73%, respectively, and they are reduced by 4.90% and 5.86%, respectively, compared with the model with whole-wavelength variables. The $R_{C}^{2}$ and $R_{CV}^{2}$ are 0.64 and 0.51, respectively, and are increased by 6.25% and 13.33%, respectively, and the RPDCV was 1.43. RPDCV < 2.5, which indicates that the predicted value of this model is not high and can only be used for rough evaluation. Moreover, RMEC and RMSECV, $R_{C}^{2}$, and $R_{CV}^{2}$ differ greatly from each other, reflecting the instability of the model.

### 2.5. Validation for Unknown Samples

**Figure 5:**
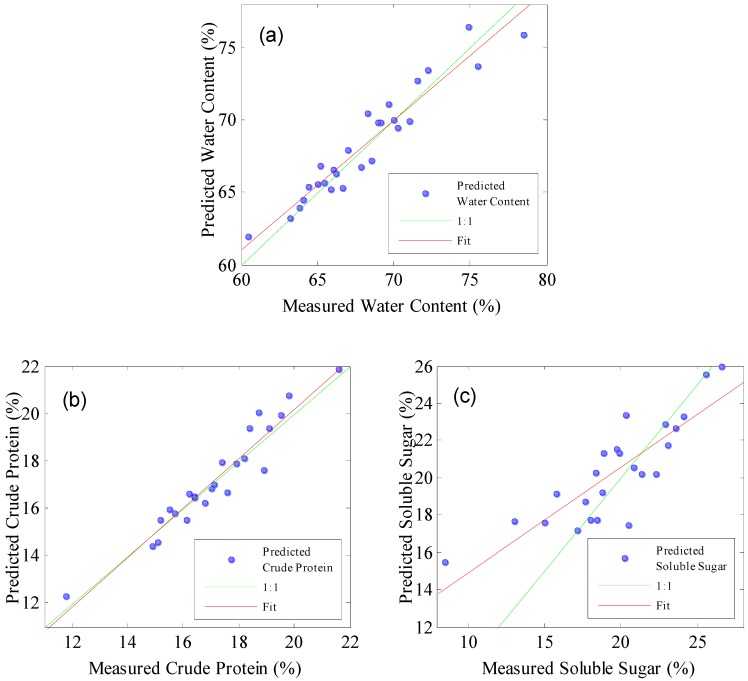
Scatter diagrams of the measured and predicted values of unknown samples; (**a**) water content in fresh mulberry leaves, (**b**) crude protein in mulberry leaves, and (**c**) soluble sugar in mulberry leaves.

The results of the verification of the unknown sample to the model are shown in Table 3. 27 unknown samples were collected to verify the predictive power of the moisture model for mulberry leaves. The results showed that $R_{P}^{2}$ and RMSEP were 0.91 and 1.18%, respectively, and the RPDP and RER were 3.43 and 15.21, respectively, $R_{P}^{2}$ was high, closer to $R_{C}^{2}$ and $R_{CV}^{2}$, RMSEP was low, similar to RMSEC and RMSECV, RPDP > 3, and RER > 10, indicating that the model is accurate and robust. The absolute error range is −2.58~2.16%, and the relative error range is −3.28~3.16% (Table 4), indicating that the model has accurate prediction ability. Figure 5a is a scatter plot of measured and predicted values for mulberry water content. This value is close to the regression line, which indicates that the model has higher prediction accuracy. Ni et al. [22] applied NIR spectroscopy and the stacked autoencoder combined with support vector regression to establish a prediction model for the moisture content of Masson pine seedling leaves. The $R_{C}^{2}$ and $R_{P}^{2}$ are 0.9946 and 0.9621, respectively, and the RMSEC and RMSEP are 0.1636 0.4249, respectively. The performance of calibration is higher than that of this study, mainly because the NIR spectrometer used is a Fourier-Transform NIR spectrometer, with a wide spectral range. However, this kind of instrument is expensive and is hard to be widely used.

Twenty four samples were collected to assess the predictive performance of the crude protein model. The $R_{P}^{2}$ and RMSEP are 0.92% and 0.61%, respectively, and the RPDP and RER are 3.34 and 16.11, respectively. The absolute error range is −1.31 to 1.36%, and the relative error range is −6.55~7.30% (Table 4). In Figure 5b, the measured and predicted values are close to the regression line. These results show that although the established model can predict crude protein of mulberry leaves, the robustness and prediction accuracy of the model are poor.

A set of 22 samples was collected to form a test set to validate the performance of the determination of soluble sugar in mulberry leaves. The $R_{P}^{2}$ is 0.71, and the RMSEP is 2.36%, the RPDP and RER are 1.29 and 4.93, respectively, RPDP < 2.5, RER < 10, and the absolute error range and relative error range between predicted and measured values are −3.04~3.33% and −14.81 to 21.09%, respectively (Table 4). In the scatter plot of the measured and predicted sugar content (Figure 5c), the measured and predicted value is not close to the regression line, which indicates that the predictive ability of the model is relatively weak, so it is difficult to predict the soluble sugar content in mulberry leaves accurately in practice. Quentin et al. [23] established a PLS prediction model for soluble sugar in spherical eucalyptus leaves by NIR spectroscopy. The R^2^ is 0.70 and RMSEP > 2.3%, which are basically consistent with the achieved results in this work. 

In this work, the accuracy of models for the determination of water content in fresh mulberry leaves is high, and the prediction accuracy for the crude protein in mulberry leaves is not as high as that of the water content, it is because the NIR spectra are sensitive to water [18]. The soluble sugar content of the mulberry leaves prediction model is not very effective, and it may because that the soluble sugar is similar to carbohydrates, polysaccharides, and cellulose, which interfere with the NIR spectra.

## 3. Conclusions

A handheld NIR spectrometer combined with chemometric methods can quickly detect the moisture in fresh mulberry leaves, as well as the crude protein and soluble sugar content in dried mulberry leaves. The detection accuracy of water and protein content was high; the RMSEPs are 0.91% and 0.92%, the RPDs are 3.43 and 3.34, respectively, and the RERs are 15.21 and 16.11, respectively. However, soluble sugar content is slightly low, and the RMSEP, RPD, and RER are 0.71%, 1.29, and 4.93, respectively. With the developed method, it will be of great importance to improve the quality of mulberry leaves for animal feeds.

## 4. Materials and Methods

### 4.1. Mulberry Leaves

Fresh mulberry leaves were collected from the mulberry resource center, the sericultural research institute, Chinese academy of agricultural sciences (Zhenjiang, Jiangsu, China). The whole leaves of the seventh or eighth position of the mulberry branch were plucked as calibration sets. The numbers of samples were 83, 77, and 80 for the calibration sets of water content, crude protein, and soluble sugar, respectively.

### 4.2. Methods

#### 4.2.1. NIR Spectra Collection

**Figure 6:**
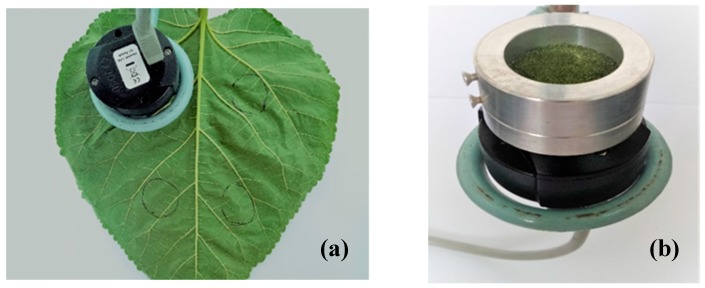
Photo of recording the NIR spectrum (**a**) fresh mulberry leaves; (**b**) mulberry leaf powders.

The NIR transflective spectra of fresh mulberry leaves were collected by a handheld NIR spectrometer (MicroNIR1700, JDSU, Santa Rosa, CA, USA). Spectra were collected from four points on each of the two superimposed samples (Figure 6a). Each point was collected three times, and the spectrometer was rotated 120° each time to collect the spectrum. A total of 12 spectra were averaged as the final spectrum of a sample.

Fresh mulberry leaves were placed in an oven and dried to constant weight at 60 °C, and then pulverized and passed through a 60-mesh sieve to obtain mulberry leaf powders. As shown in Figure 6b, 1.5~2 cm high of mulberry leaf powder was poured in the drum (the bottom of which is the window of the NIR spectrometer) to collect the NIR diffuse reflectance spectrum of the mulberry leaf powder. Each sample was collected three times, and the sample was rotated 120° each time to collect the spectrum, an average of the three spectra was used as the final spectrum of a sample.

For the spectral acquisition parameters, the spectral range was 950~1650 nm, and the spectral resolution was 12.5 nm (at 1000 nm), the number of scans was 50, and the integration time was 15 ms. As a reference, a 99% Spectralon reflection standard (Labsphere, Inc., North Sutton, NA, USA) was used, all measurements were performed at room temperature and relative humidity of 35–40%.

#### 4.2.2. Reference Determination

The water content of fresh mulberry leaves was determined by the drying method at 105 °C. The crude protein and soluble sugar in dry mulberry leaves were determined by the Kjeldahl method [24] and the anthrone-sulfuric acid colorimetric method [25], respectively. Each component was subjected to three parallel determinations, and the average value was used as the final result.

#### 4.2.3. Spectra Pretreatment

Collected NIR spectra contain not only the component information of the sample but also interference information such as stray light, baseline drift, background noise, etc., which can reduce the reliability and stability of the spectral model. This may be due to the rough surface of leaves, an abundance of veins on fresh mulberry leaves, and uneven mulberry leaf particles. In this work, spectral data were pretreated using different combinations of the 1st Der, SNV, mean center, and autoscaling to eliminate interfering information and to highlight spectral information.

#### 4.2.4. Wavelength Selection

Generally, there is redundant information in the raw NIR spectra. Therefore, when the prediction model is built with the whole wavelength variables, the accuracy of the model will be reduced. The wavelength optimization can extract the characteristic wavelength variables of the component in samples to establish a more reliable prediction model [26]. At present, the commonly used characteristic wavelength screening algorithms mainly include genetic algorithms [27], CARS, UVE, moving window, and RF. In this work, the UVE, CARS, and RF methods were used to improve the reliability and accuracy of the prediction model.

The CARS is based on the simple and effective “survival of the fittest” principle to select wavelength variables, and it selects the optimal combination of wavelength variables with larger absolute regression coefficients in PLS regression [28]. The UVE is a wavelength optimization method based on the PLS regression coefficient *b* to eliminate the useless information of spectral data [14]. RF is a novel feature wavelength optimization method that can be iteratively modeled with a small number of wavelength variables [29,30]. This algorithm can calculate the probability that each variable is selected, and the wavelength is preferred according to the magnitude of the probability.

#### 4.2.5. PLS Calibration

PLS is a linear regression modeling method for multiple independent variables versus multiple dependent variables [31,32,33]. It was used in calibration in this work.

#### 4.2.6. Evaluation Method

The evaluation indicators for the model mainly include RMSEC, RMSECV, $R_{C}^{2}$, and $R_{CV}^{2}$. The smaller the values of RMSEC and RMSECV and the closer they are to each other, the more the prediction accuracy and the higher the stability of the model. The R^2^ is used to describe the correlation between the two group variables. In the prediction model, the R^2^ between the predicted and the measured values has a value range of 0~1, the closer the R^2^ is to 1, the closer the predicted value is to the actual value. RPD is the ratio of SD to RMSE for the prediction set [34]. The higher the RPD value, the better the prediction ability of the established model. When RPD ≥ 3, it indicates that the prediction model has a good effect and can be used for rapid analysis and detection of unknown samples. When 2.5 < RPD <3, it indicates that the prediction model has general analysis ability, and the prediction accuracy needs to be improved. When RPD < 2.5, the prediction model is difficult. Rapid detection and analysis of unknown samples. RER is the ratio of the reference range of the prediction set to the RMSEP, which is similar in nature to RPD, but at least higher than 10 indicates that the prediction model is reliable.

#### 4.2.7. Validation With Unknown Samples

Unknown mulberry leaves were collected to validate the prediction capability of built models for the water content, crude protein, and soluble sugar.

#### 4.2.8. Software

The UVE was run on the toolbox of Chemoactbx, and the CARS and RF algorithms were performed with the libPLS toolbox (http://www.libpls.net/) [28], and they were all run on the MATLAB R2009 (MathWorks, Natick, MA, USA).

## Figures and Tables

**Table 1:** Statistical results of reference values of the components in mulberry leaves.

Samples	Components (%)	Data Set	Number	Min	Max	Mean	Range	SD
Fresh mulberry leaves	Water content	Total	110	60.44	78.46	68.24	18.02	3.75
		Cal	83	60.58	77.81	68.28	17.22	3.66
		Pre	27	60.44	78.46	68.14	18.02	4.06
Dry mulberry leaves	Crude protein	Total	101	11.10	23.50	17.41	12.40	2.27
		Cal	77	11.10	23.50	17.50	12.40	2.34
		Pre	24	11.80	21.60	17.14	9.80	2.03
	Soluble sugar	Total	104	8.47	31.01	19.97	22.54	3.92
		Cal	80	8.55	31.01	20.09	22.46	3.91
		Pre	24	8.47	26.62	19.59	18.15	4.04

**Table 2:** The influence of pretreatment methods on the performance of model prediction accuracy.

Components	Pretreatment Method	LVs	RMSEC (%)	$R_{C}^{2}$	RMSECV (%)	$R_{CV}^{2}$
Water content	None	7	1.18	0.89	1.34	0.86
	1st Der+ mean center	7	1.09	0.91	1.32	0.87
	SNV+ mean center	7	1.04	0.92	1.21	0.89
	autoscaling	7	1.23	0.89	1.46	0.84
	1st Der+ SNV+ mean center	7	1.06	0.92	1.25	0.88
	1st Der+ autoscaling	7	1.19	0.89	1.38	0.86
	SNV+ autoscaling	7	1.08	0.91	1.24	0.88
	**1st Der+ SNV+ autoscaling**	**7**	**1.00**	**0.92**	**1.17**	**0.90**
Crude protein	None	9	0.89	0.85	1.11	0.78
	SNV+ mean center	8	0.82	0.88	1.00	0.82
	1st Der+ mean center	9	0.85	0.87	1.08	0.79
	autoscaling	9	0.88	0.86	1.10	0.78
	1st Der+ SNV+ mean center	8	0.80	0.88	0.97	0.83
	1st Der+ autoscaling	8	0.86	0.86	1.05	0.80
	SNV+ autoscaling	8	0.76	0.89	0.97	0.83
	**1st Der+ SNV+ autoscaling**	**8**	**0.74**	**0.90**	**0.97**	**0.83**
Soluble sugar	None	9	2.56	0.56	3.21	0.35
	SNV+ mean center	8	2.52	0.58	3.33	0.32
	1st Der+ mean center	8	2.43	0.61	3.14	0.37
	autoscaling	9	2.53	0.57	3.30	0.33
	1st Der+ SNV+ mean center	8	2.53	0.58	3.18	0.36
	1st Der+ autoscaling	8	2.52	0.58	3.09	0.40
	SNV+ autoscaling	7	2.54	0.57	3.08	0.39
	**1st Der+ SNV+ autoscaling**	**7**	**2.45**	**0.60**	**2.90**	**0.45**

**Table 3:** Model results of mulberry leaf wavelength optimization.

Components	Methods	LVs	RMSEC (%)	$R_{C}^{2}$	RMSECV (%)	$R_{CV}^{2}$	RMSEP (%)	$R_{P}^{2}$	RPDCV	RPDR	RER
Water content	PLS	7	1.00	0.92	1.17	0.90	1.22	0.91	3.14	3.33	14.80
	CARS-PLS	7	0.95	0.93	1.11	0.91	1.21	0.91	3.30	3.36	14.93
	UVE-PLS	7	0.94	0.93	1.10	0.91	1.19	0.91	3.34	3.41	15.12
	**RF-PLS**	**7**	**0.96**	**0.93**	**1.13**	**0.90**	**1.18**	**0.91**	**3.25**	**3.43**	**15.21**
Crude protein	PLS	8	0.74	0.90	0.97	0.83	0.67	0.89	2.42	3.02	14.56
	**CARS-PLS**	**9**	**0.71**	**0.91**	**0.97**	**0.83**	**0.61**	**0.92**	**2.43**	**3.34**	**16.11**
	UVE-PLS	7	0.73	0.90	0.86	0.86	0.64	0.91	2.74	3.19	15.39
	RF-PLS	7	0.74	0.90	0.88	0.86	0.65	0.90	2.67	3.11	14.99
Soluble sugar	PLS	7	2.45	0.60	2.90	0.45	2.57	0.64	1.35	1.19	4.54
	CARS-PLS	9	2.32	0.64	2.84	0.48	2.37	0.72	1.38	1.28	4.92
	**UVE-PLS**	**8**	**2.33**	**0.64**	**2.73**	**0.51**	**2.36**	**0.71**	**1.43**	**1.29**	**4.93**
	RF-PLS	10	2.27	0.66	2.84	0.48	2.40	0.71	1.38	1.27	4.86

**Table 4:** Verification results for unknown samples with the built models.

Comp Onents	No.	Measured Value	Predicted Value	Absolute Error	Relative Error	No.	Measured Value	Predicted Value	Absolute Error	Relative Error
Water content (*n* = 27)	**W1**	60.44	61.94	1.50	2.48	**W15**	68.32	70.48	2.16	3.16
	**W2**	63.22	63.19	−0.03	−0.04	**W16**	68.53	67.19	−1.35	−1.97
	**W3**	63.84	63.98	0.14	0.21	**W17**	68.95	69.83	0.88	1.28
	**W4**	64.07	64.43	0.37	0.57	**W18**	69.14	69.78	0.65	0.93
	**W5**	64.44	65.42	0.98	1.53	**W19**	69.62	71.12	1.50	2.15
	**W6**	65.06	65.52	0.46	0.71	**W20**	70.00	69.99	0.00	0.00
	**W7**	65.18	66.80	1.62	2.49	**W21**	70.23	69.46	−0.77	−1.10
	**W8**	65.48	65.69	0.21	0.33	**W22**	71.04	69.88	−1.16	−1.63
	**W9**	65.87	65.21	−0.66	−1.00	**W23**	71.50	72.71	1.21	1.69
	**W10**	66.01	66.54	0.53	0.80	**W24**	72.22	73.45	1.23	1.70
	**W11**	66.26	66.30	0.04	0.05	**W25**	74.88	76.41	1.53	2.04
	**W12**	66.64	65.27	−1.37	−2.05	**W26**	75.49	73.74	−1.75	−2.32
	**W13**	66.97	67.95	0.99	1.48	**W27**	78.46	75.89	−2.58	−3.28
	**W14**	67.86	66.75	−1.11	−1.63					
Crude protein (*n* = 20)	**P1**	11.80	12.29	0.49	4.13	**P13**	17.10	16.98	−0.12	−0.70
	**P2**	14.90	14.41	−0.49	−3.29	**P14**	17.40	17.94	0.54	3.12
	**P3**	15.10	14.58	−0.52	−3.43	**P15**	17.60	16.67	−0.93	−5.28
	**P4**	15.20	15.52	0.32	2.08	**P16**	17.90	17.87	−0.03	−0.19
	**P5**	15.50	15.94	0.44	2.85	**P17**	18.20	18.08	−0.12	−0.63
	**P6**	15.70	15.79	0.09	0.60	**P18**	18.40	19.37	0.97	5.26
	**P7**	16.10	15.48	−0.62	-3.83	**P19**	18.70	20.06	1.36	7.30
	**P8**	16.20	16.63	0.43	2.63	**P20**	18.90	17.59	−1.31	−6.95
	**P9**	16.40	16.45	0.05	0.33	**P21**	19.10	19.39	0.29	1.52
	**P10**	16.40	16.48	0.08	0.51	**P22**	19.50	19.92	0.42	2.16
	**P11**	16.80	16.24	−0.56	−3.34	**P23**	19.80	20.74	0.94	4.73
	**P12**	17.00	16.82	−0.18	−1.04	**P24**	21.60	21.85	0.25	1.16
Soluble sugar (*n* = 21)	**S1**	14.97	17.58	2.61	17.43	**S12**	20.35	23.35	3.00	14.74
	**S2**	15.79	19.12	3.33	21.09	**S13**	20.52	17.48	−3.04	−14.81
	**S3**	17.15	17.14	−0.01	−0.06	**S14**	20.84	20.53	−0.31	−1.49
	**S4**	17.69	18.69	1.00	5.65	**S15**	21.31	20.20	−1.11	−5.21
	**S5**	18.02	17.75	−0.27	−1.50	**S16**	22.32	20.21	−2.11	−9.45
	**S6**	18.32	20.26	1.94	10.59	**S17**	22.88	22.84	−0.04	−0.17
	**S7**	18.44	17.76	−0.68	−3.69	**S18**	23.05	21.72	−1.33	−5.77
	**S8**	18.73	19.20	0.47	2.51	**S19**	23.56	22.69	−0.87	−3.69
	**S9**	18.87	21.29	2.42	12.82	**S20**	24.08	23.28	−0.80	−3.32
	**S10**	19.73	21.55	1.82	9.22	**S21**	25.52	25.58	0.06	0.24
	**S11**	19.91	21.34	1.43	7.18	**S22**	26.62	25.98	−0.64	−2.40

## References

[1] Al-Kirshi R.A., Alimon A., Zulkifli I., Atefeh S., Zahari M.W., Ivan M. (2013). Nutrient digestibility of mulberry leaves (*Morus Alba*). Ital. J. Anim. Sci..

[2] Panja P. (2013). The effects of dietary mulberry leaves (*Morus alba* L.) on chicken performance, carcass, egg quality and cholesterol content of meat and egg. Walailak J. Sci. Technol..

[3] Huyen N.T., Wanapat M., Navanukraw C. (2012). Effect of mulberry leaf pellet (MUP) supplementation on rumen fermentation and nutrient digestibility in beef cattle fed on rice straw-based diets. Anim. Feed Sci. Technol..

[4] Hiromitsu N., Shinji O., Eriko K., Sukunya C., Midori T., Hajime K., Rensuke K. (2011). Effect of environmental conditions on the α-glucosidase inhibitory activity of mulberry leaves. J. Agric. Chem. Soc. Jpn..

[5] Islam M., Siddiqui M.N., Khatun A., Siddiky M., Rahman M., Bostami A., Selim A. (2014). Dietary effect of mulberry leaf (*Morus alba*) meal on growth performance and serum cholesterol level of broiler chickens. SAARC J. Agric..

[6] Zhu Z., Jiang J.J., Jie Y.U., Mao X.B., Bing Y.U., Chen D.W. (2019). Effect of dietary supplementation with mulberry (*Morus alba* L.) leaves on the growth performance, meat quality and antioxidative capacity of finishing pigs. J. Integr. Agric..

[7] Naranjo A.A., García J.A., Esperance M. (2017). Partial or total replacement of commercial concentrate with on-farm-grown mulberry forage: Effects on lamb growth and feeding costs. Trop. Anim. Health Prod..

[8] Dasappa D., Ramaswamy S. (2006). Efficacy of cyanobacterial biofertilizer (CBB) on leaf yield and quality of mulberry and its impact on silkworm cocoon characters. Int. J. Ind. Entomol..

[9] Li-chan E.C.Y., Griffiths P.R., Chalmers J.M. (2010). Applications of Vibrational Spectroscopy in Food Science: Volume I: Instrumentation and Fundamental Applications.

[10] Osborne B.G., Kays S.E., Barton F.E., Cozzolino D., Cattaneo T.M.P. (2006). Near-Infrared Spectroscopy in Food Science and Technology.

[11] Zhang Y., Dong Y., Xiang B., Xu J. (2015). Feasibility research on rapid detection of prochloraz in green tea soft drink by near-infrared spectroscopy. Food Anal. Method.

[12] Toledo-Martín E., García-García M., Font R., Moreno-Rojas J., Salinas-Navarro M., Gómez P., del Río-Celestino M. (2018). Quantification of total phenolic and carotenoid content in blackberries (*Rubus Fructicosus* L.) using near infrared spectroscopy (NIRS) and multivariate analysis. Molecules.

[13] Yang Z., Nie G., Pan L., Zhang Y., Huang L., Ma X., Zhang X. (2017). Development and validation of near-infrared spectroscopy for the prediction of forage quality parameters in *Lolium multiflorum*. PeerJ.

[14] Yan H., Han B.X., Wu Q.Y., Jiang M.Z., Gui Z.Z. (2011). Rapid detection of *Rosa laevigata* polysaccharide content by near-infrared spectroscopy. Spectrochim. Acta Part A.

[15] Yan H., Siesler H.W. (2018). Quantitative analysis of a pharmaceutical formulation: Performance comparison of different handheld near-infrared spectrometers. J. Pharm. Biomed. Anal..

[16] Swart E., Brand T., Engelbrecht J. (2012). The use of near infrared spectroscopy (NIRS) to predict the chemical composition of feed samples used in ostrich total mixed rations. S. Afr. J. Anim. Sci..

[17] Tahir M., Shim M.Y., Ward N.E., Westerhaus M.O., Pesti G.M. (2012). Evaluation of near-infrared reflectance spectroscopy (NIRS) techniques for total and phytate phosphorus of common poultry feed ingredients. Poult. Sci..

[18] Cozzolino D., Labandera M. (2002). Determination of dry matter and crude protein contents of undried forages by near-infrared reflectance spectroscopy. J. Sci. Food Agric..

[19] Neves M.D.G., Poppi R.J., Siesler H.W. (2019). Rapid Determination of nutritional parameters of pasta/sauce blends by handheld near-infrared spectroscopy. Molecules.

[20] Li H., Liang Y., Xu Q., Cao D. (2009). Key wavelengths screening using competitive adaptive reweighted sampling method for multivariate calibration. Anal. Chim. Acta.

[21] Li X., Sun C., Luo L., He Y. (2015). Determination of tea polyphenols content by infrared spectroscopy coupled with iPLS and random frog techniques. Comput. Electron. Agric..

[22] Ni C., Zhang Y., Wang D. (2018). Moisture content quantization of masson pine seedling leaf based on stacked autoencoder with near-infrared spectroscopy. J. Electr. Comput. Eng..

[23] Quentin A., Rodemann T., Doutreleau M., Moreau M., Davies N. (2017). Application of near-infrared spectroscopy for estimation of non-structural carbohydrates in foliar samples of *Eucalyptus globulus* Labilladière. Tree Physiol..

[24] Granados-Chinchilla F., Rodríguez C. (2014). Bioavailability of in-feed tetracyclines is influenced to a greater extent by crude protein rather than calcium. Anim. Feed Sci. Technol..

[25] Ibrahim M.H., Jaafar H.Z., Karimi E., Ghasemzadeh A. (2013). Impact of organic and inorganic fertilizers application on the phytochemical and antioxidant activity of kacip fatimah (*Labisia pumila* Benth). Molecules.

[26] Yun Y.H., Liang Y.Z., Xie G.X., Li H.D., Cao D.S., Xu Q.S. (2013). A perspective demonstration on the importance of variable selection in inverse calibration for complex analytical systems. Analyst.

[27] Rady A.M., Guyer D.E. (2015). Evaluation of sugar content in potatoes using NIR reflectance and wavelength selection techniques. Postharvest Biol. Technol..

[28] Li H.D., Xu Q.S., Liang Y.Z. (2018). libPLS: An integrated library for partial least squares regression and linear discriminant analysis. Chemom. Intell. Lab. Syst..

[29] Yun Y.H., Li H.D., Wood L.R., Fan W., Wang J.J., Cao D.S., Xu Q.S., Liang Y.Z. (2013). An efficient method of wavelength interval selection based on random frog for multivariate spectral calibration. Spectrochim. Acta Part A.

[30] Li H.D., Xu Q.S., Liang Y.Z. (2012). Random frog: An efficient reversible jump Markov Chain Monte Carlo-like approach for variable selection with applications to gene selection and disease classification. Anal. Chim. Acta.

[31] Xue J., Yang Q., Yun J., Liu Y., Wan G. (2016). Rapid determination of puerarin by near-infrared spectroscopy during percolation and concentration process of puerariae lobatae radix. Pharmacogn. Mag..

[32] Katarzyna W.O., Igor K., Ewa S. (2018). Evaluation of quality parameters of apple juices using near-infrared spectroscopy and chemometrics. J. Spectrosc..

[33] Nascimento P.A.M., Carvalho L.C.D., Júnior L.C.C., Pereira F.M.V. (2016). Robust PLS models for soluble solids content and firmness determination in low chilling peach using near-infrared spectroscopy (NIR). Postharvest Biol. Technol..

[34] Martinez-Valdivieso D., Font R., Gomez P., Blanco-Diaz T., Del Rio-Celestino M. (2014). Determining the mineral composition in *Cucurbita pepo* fruit using near infrared reflectance spectroscopy. J. Sci. Food Agric..

